# Comparative Meta-Analysis of Tenofovir Disoproxil Fumarate versus Emtricitabine and Tenofovir Disoproxil Fumarate as Treatments for Patients with Chronic Hepatitis B

**DOI:** 10.1038/srep11854

**Published:** 2015-07-13

**Authors:** Guangying Cui, Xuejun Xu, Hongyan Diao

**Affiliations:** 1 State Key Laboratory for Diagnosis and Treatment of Infectious Diseases, The First Affiliated Hospital, School of Medicine, Zhejiang University, Hangzhou, Zhejiang, 310003, China; 2 Collaborative Innovation Center for Diagnosis and Treatment of Infectious Diseases, Hangzhou, China; 3 Department of Orthodontics, Affiliated Stomatology Hospital, School of Medicine, Zhejiang University, Hangzhou, Zhejiang, 310006, China

## Abstract

Tenofovir disoproxil fumarate (TDF) monotherapy has proven superior antiviral efficacy in chronic hepatitis B (CHB) patients; however, whether the combination of TDF and emtricitabine (FTC) exerts a significant advantage remains controversial. A meta-analysis was performed to comprehensively compare the therapeutic effects of FTC/TDF combination with TDF alone in CHB patients. Five studies involving 614 patients were identified, and subgroup analysis was performed based on the nucleos(t)ide treatment history. Our results revealed that in patients with nucleos(t)ide-naïve treatment, there were no significant differences between the treatment groups with TDF alone and FTC/TDF combination after 12 and 24 weeks; however, the FTC/TDF combination showed better viral suppression efficacy versus TDF alone after 48 (OR = 2.16, 95% CI = 1.06–4.41, P = 0.03), 96 (OR = 2.76, 95% CI = 1.29–5.92, P = 0.009) and 192 weeks (OR = 2.60, 95% CI = 1.21–5.56, P = 0.01). In patients with nucleos(t)ide treatment history, no differences were noted between the two treatment groups after 12, 24, 48 and 96 weeks. Our results indicated that FTC/TDF combination showed better viral suppression efficacy versus TDF alone in CHB patients with nucleos(t)ide-naïve treatment, while both treatments provided similar viral suppression efficacy in CHB patients with nucleos(t)ide treatment history.

Hepatitis B virus (HBV) infection remains a major global health problem. It is a leading cause of chronic liver disease and chronic infection that may progress to liver cirrhosis and liver cancer^1^,^2^. There are more than 240 million people with chronic (long-term) liver infections, and more than 780000 people die from the acute or chronic consequences of hepatitis B every year^3^. The major goal of drug treatment for patients with chronic hepatitis B (CHB) is to improve quality of life and survival by preventing progression of the disease to cirrhosis, decompensated cirrhosis, end-stage liver disease, liver cancer and death^4^,^5^. This goal can be achieved if HBV replication can be suppressed in a sustained manner^5^.

CHB patients are treated mainly with interferon injections and oral antiviral agents, such as nucleotide analogues^3^. As the first nucleotide analogue approved for the treatment of CHB infection, lamivudine (LAM) remains a widely prescribed oral antiviral agent worldwide, especially in Asia, due to its widespread availability, relative low toxicity and cost^6^. Although LAM is effective in suppressing viral replication and patients’ tolerance, the long-term administration has been limited by the high rate of LAM resistance^5^,^7^. As another anti-HBV therapy, adefovir dipivoxil (ADV) is effective in the setting of naïve and LAM-resistant patients^8^,^9^ and is also indicated in patients with decompensated liver disease^10^. However, the key limitation of ADV is a relatively slow rate of viral decline^10^, and long-term ADV monotherapy can also induce a high rate of ADV-resistance mutations in HBeAg-negative chronically infected HBV patients^8^.

Tenofovir disoproxil fumarate (TDF) is a nucleotide analogue closely related to ADV. In 5-year clinical trials conducted in naïve CHB patients, TDF treatment showed well-tolerated and produced a significant improvement in hepatic fibrosis, durable suppression of HBV replication and delayed development of resistance^8^,^11^. Importantly, TDF has also been indicated to be highly efficacious in patients with LAM-resistant HBV infection and even in patients after failure of ADV therapy^12^,^13^. A retrospective study demonstrated that TDF retains significant activity against HBV in heavily pretreated patients with a high rate of genotypic resistance mutations^14^. In short, TDF has proven superior antiviral efficacy in the CHB patients^8^,^11^,^15^.

Emtricitabine (FTC) is a cytosine nucleoside analogue approved for use in human immunodeficiency virus (HIV) infection, and has nearly identical phenotypic resistance profiles and the same biological cutoff of LAM^16^. The combination of TDF and FTC is approved for the treatment of HIV infection. Both TDF and FTC showed activity against HBV^15^,^17^, and an *in vitro* study suggested that the combination of TDF and FTC could produce a synergistic effect in term of anti-HBV activity^18^. Some clinical trials were performed to verify the treatment efficacy of the FTC/TDF combination 10,19,20,21,22. However, whether the combination of TDF and FTC provides a significantly predominant advantage over TDF monotherapy in CHB patients remains controversial.

In the present study, we performed a systematic review and meta-analysis to qualitatively and quantitatively compare the therapeutic effect of TDF alone with FTC/TDF combination in CHB patients. We also performed a statistical analysis of secondary outcomes, such as subgroup variation, serological responses and safety assessment.

## Results

### Study selection and characteristics

**Figure 1:**
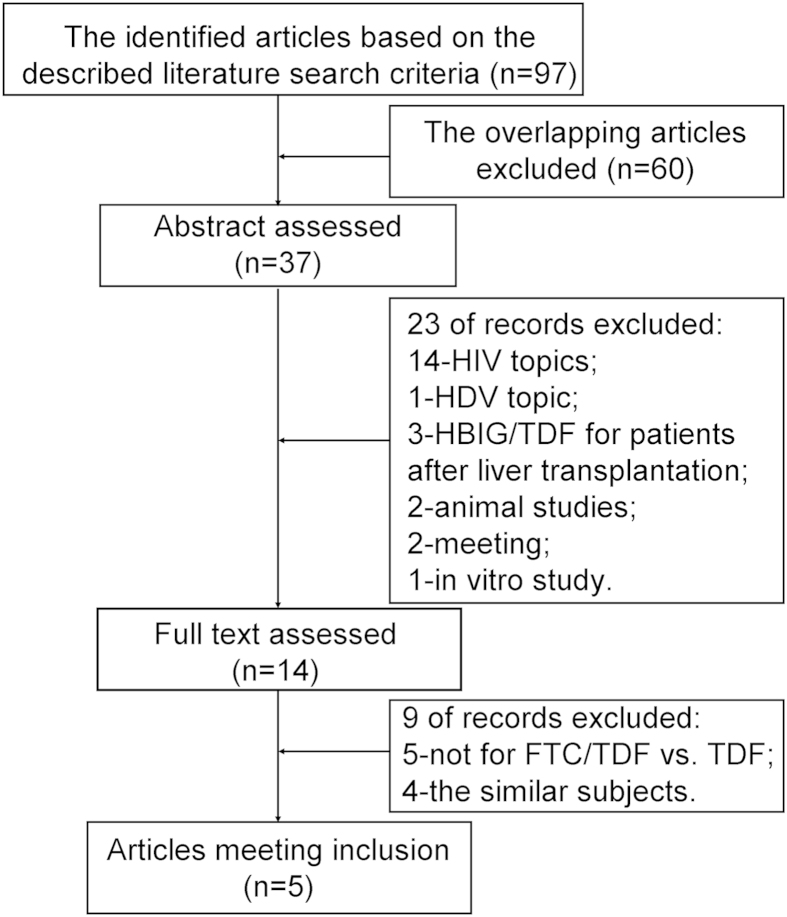
Flow diagram of literature search strategies.

As shown in Fig. 1, 97 potentially eligible studies were screened out in the preliminary search. Of these, 60 articles were excluded based on overlapping articles obtained from the different databases, and 28 articles were excluded due to their improper titles and abstracts. Of the remaining nine articles, four articles were eliminated because the same trials were reported more than once. Ultimately, after detailed and sufficient evaluation, five studies^10^,^19^,^20^,^21^,^22^ were found to meet our inclusion criteria and were retrieved for further analysis. A flowchart of the study selection procedure is displayed in Fig. 1.

Table 1 summarizes the characteristics of the five studies involving 614 patients. Four studies^10^,^20^,^21^,^22^ with 601 patients were multicenter, double-blind, randomized controlled trials (RCTs). The quality of these four studies scored 4 and another non-RCT study scored 1 using a Jadad evaluation system^23^. Of the five studies, there were no significant differences between the treatment groups with TDF alone and FTC/TDF combination with regard to demographics and characteristics of CHB patients including age, gender, HBV DNA copies, HBeAg positive patients, normal ALT patients and study duration. In term of nucleos(t)ide treatment history, a total of 488 patients in the four studies^10^,^19^,^20^,^21^ had received nucleos(t)ide drugs including ADV or LAM, while another study^22^ enrolled 126 nucleos(t)ide-naïve patients with high HBV viral load; thus, the whole analysis was stratified by the nucleos(t)ide drug treatment history.

### Primary outcome: viral suppression efficacy

**Figure 2:**
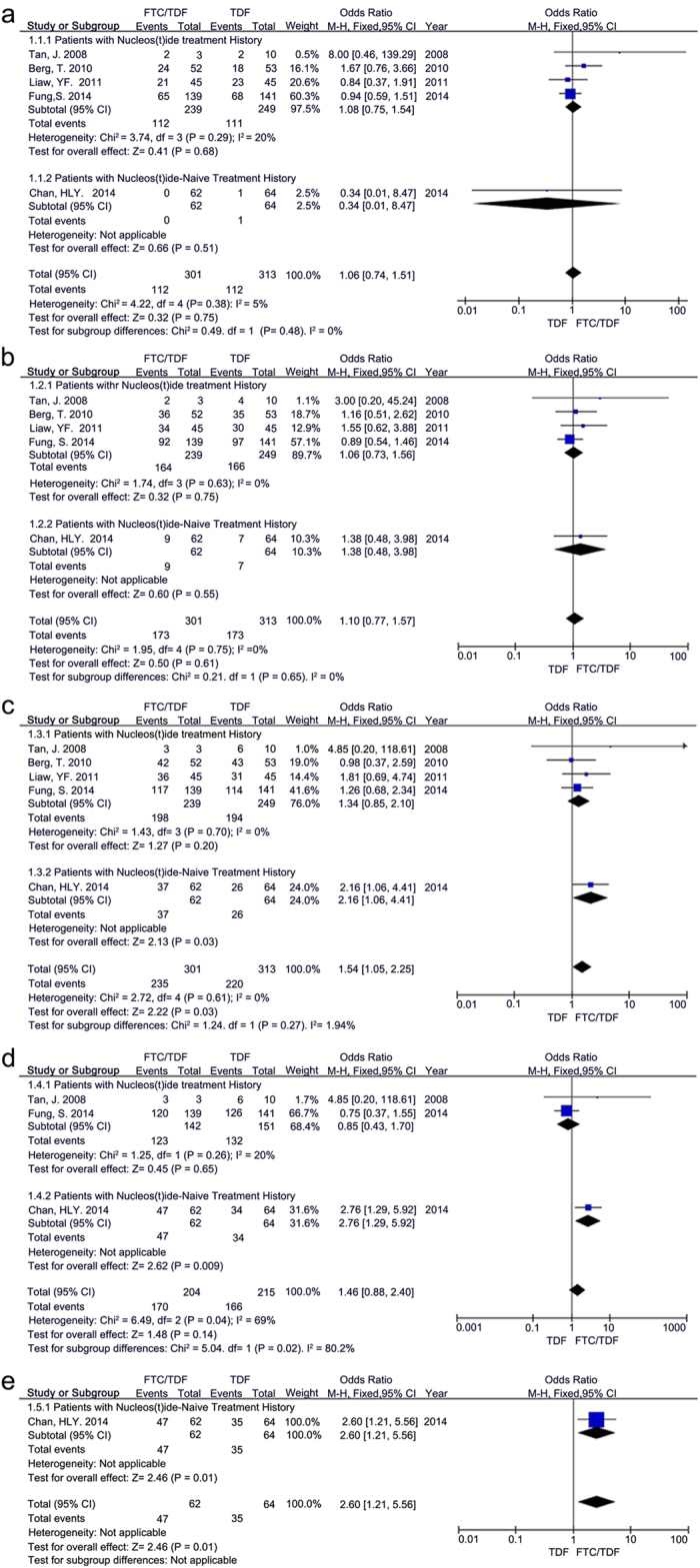
Forest plot displaying the primary outcomes of viral suppression efficacy for CHB patients at different time points after treatments with TDF alone and FTC/TDF combination, respectively. Forest plot showed viral suppression efficacy for CHB patients with nucleos(t)ide-(or naïve) treatment history after (**a**) 12 weeks, (**b**) 24 weeks, (**c**) 48 weeks, (**d**) 96 weeks and (**e**) 192 weeks of treatments with TDF alone and FTC/TDF combination, respectively.

The forest plot in Fig. 2 showed the results of viral suppression efficacy for CHB patients after treatment with TDF alone or with FTC/TDF combination. The forest plots showed that there was no statistically significant heterogeneity among the studies in each subgroup; therefore, the fixed effect model was adopted in the analysis. The total analysis of the five studies for comparison of TDF alone and FTC/TDF combination showed no significant differences after 12 weeks (Fig. 2a, OR = 1.06, 95% CI = 0.74–1.51, P = 0.75, I^2^ = 5%) and 24 weeks (Fig. 2b, OR = 1.10, 95% CI = 0.77–1.57, P = 0.61, I^2^ = 0%), but an obvious difference was observed after 48 weeks (Fig. 2c, OR = 1.54, 95% CI = 1.05–2.25, P = 0.03, I^2^ = 0%). Notably, the total analysis showed statistically significant heterogeneity and no obvious difference between the two treatment groups after 96 weeks (Fig. 2d, OR = 1.46, 95% CI = 0.88–2.40, P = 0.14, I^2^ = 69%), but an obvious difference between the two treatment groups after 192 weeks (Fig. 2e, OR = 2.60, 95% CI = 1.21–5.56, P = 0.01). We performed the subgroup analysis to clarify the ambiguity in these results.

When evaluated according to nucleos(t)ide drug treatment history (prespecified subgroup analysis), no differences were observed between the treatment groups with TDF alone and FTC/TDF combination in patients with nucleos(t)ide treatment history after 12 weeks (Fig. 2a, OR = 1.08, 95% CI = 0.75–1.54, P = 0.68, I^2^ = 20%), 24 weeks (Fig. 2b, OR = 1.06, 95% CI = 0.73–1.56, P = 0.75, I^2^ = 0%), 48 weeks (Fig. 2c, OR = 1.34, 95% CI = 0.85–2.10, P = 0.20, I^2^ = 0%), and 96 weeks (Fig. 2d, OR = 0.85, 95% CI = 0.43–1.70, P = 0.65, I^2^ = 20%).

In patients with nucleos(t)ide-naïve treatment history, the same outcome was observed in the two treatment groups after 12 weeks (Fig. 2a, OR = 0.34, 95% CI = 0.01–8.47, P = 0.51) and 24 weeks (Fig. 2b, OR = 1.38, 95% CI = 0.48–3.98, P = 0.55). However, treatment with FTC/TDF combination showed better viral suppression efficacy versus treatment with TDF alone after 48 weeks (Fig. 2c, OR = 2.16, 95% CI = 1.06–4.41, P = 0.03), 96 weeks (Fig. 2d, OR = 2.76, 95% CI = 1.29–5.92, P = 0.009) and 192 weeks (Fig. 2e, OR = 2.60, 95% CI = 1.21–5.56, P = 0.01).

Based on the above OR, 95% CI and P-values, treatment with TDF alone and with FTC/TDF combination showed similar viral suppression efficacy in CHB patients with nucleos(t)ide treatment history. However, in CHB patients with nucleos(t)ide-naïve treatment history, treatment with FTC/TDF combination provided better viral suppression efficacy versus treatment with TDF alone.

### Secondary outcomes: serological responses

**Figure 3:**
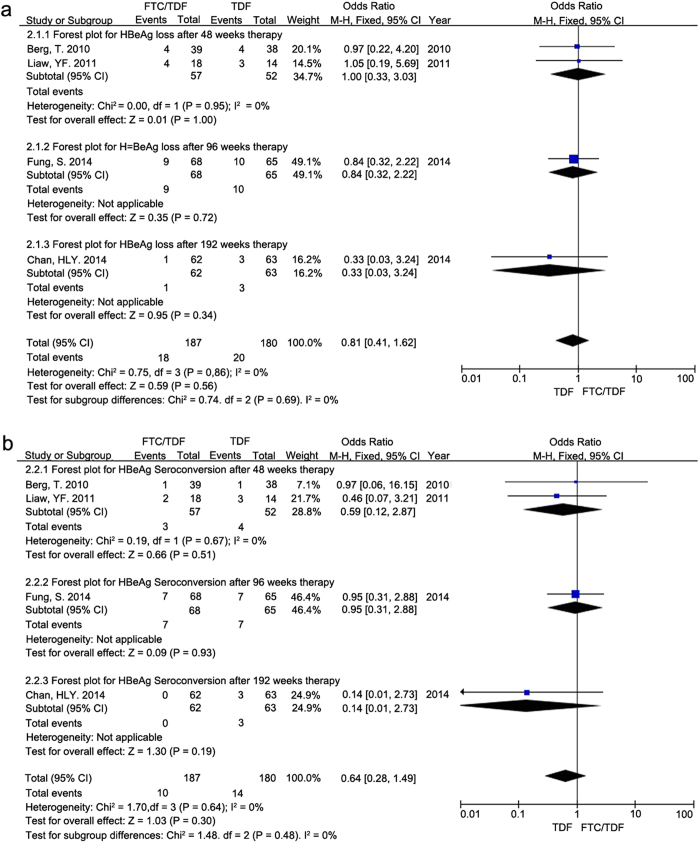
Forest plot displaying the secondary outcomes of serological responses for CHB patients at different time points after treatments with TDF alone and FTC/TDF combination, respectively. Forest plots showed the comparison of (**a**) HBeAg loss and (**b**) HBeAg seroconversion in CHB patients after 48, 96 and 192 weeks of treatments with TDF alone and FTC/TDF combination, respectively.

Serological responses mainly included HBeAg loss and HBeAg seroconversion in CHB patients after treatment with TDF alone or with FTC/TDF combination. Fig. 3a showed a comparison of HBeAg loss between the treatment groups with TDF alone and FTC/TDF combination at different time points after therapy. There were no significant differences between the two treatment groups with regards to HBeAg loss after 48 weeks (OR = 1.00, 95% CI = 0.33−3.03, P = 1.00, I^2^ = 0%), 96 weeks (OR = 0.84, 95% CI = 0.32–2.22, P = 0.72), and 192 weeks (OR = 0.33, 95% CI = 0.03–3.24, P = 0.34).

A comparison of HBeAg seroconversion between the two treatment groups at different time points after therapy was shown in Fig. 3b. Both treatments with TDF alone and FTC/TDF combination showed similar efficacy in term of HBeAg seroconversion in CHB patients after 48 weeks (OR = 0.59, 95% CI = 0.12–2.87, P = 0.51, I^2^ = 0%), 96 weeks (OR = 0.95, 95% CI = 0.31–2.88, P = 0.93), and 192 weeks (OR = 0.14, 95% CI = 0.01–2.73, P = 0.19).

### Secondary outcomes: safety assessment

The safety of both treatments with TDF alone and FTC/TDF combination in CHB patients was assessed with regard to overall adverse events (AE), drug-related AE, serious adverse events (SAE) and drug-related SAE.

**Figure 4:**
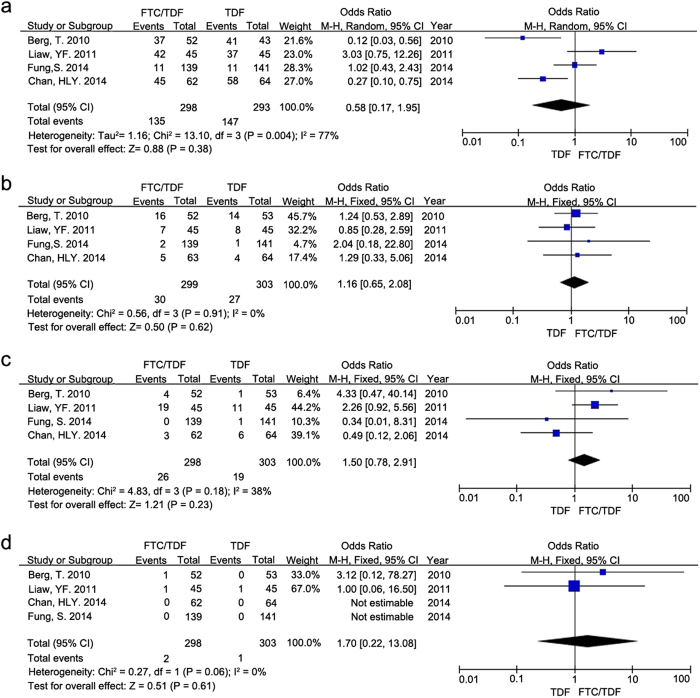
Forest plot displaying the secondary outcomes of safety assessment for CHB patients after treatments with TDF alone and FTC/TDF combination, respectively. Forest plot showed the comparison of (**a**) overall AE, (**b**) drug-related AE, (**c**) SAE and (**d**) drug-related SAE for CHB patients after treatments with TDF alone and FTC/TDF combination, respectively.

The total analysis for comparison of overall AE between the treatment groups with TDF alone and FTC/TDF combination showed no obvious difference with regard to overall AE (Fig. 4a, OR = 0.58, 95% CI = 0.17–1.95, P = 0.38, I^2^ = 77%, the random effect model). Furthermore, there was no significant difference between the two treatment groups with regard to drug-related AE (Fig. 4b, OR = 1.16, 95% CI = 0.65–2.08, P = 0.62, I^2^ = 0%).

Moreover, the analysis of SAE between the two treatment groups indicated no obvious differences (Fig. 4c, OR = 1.50, 95% CI = 0.78–2.91, P = 0.23, I^2^ = 38%). Also, there was no significant difference between the two treatment groups with regard to drug-related SAE (Fig. 4d, OR = 1.70, 95% CI = 0.22–13.08, P = 0.61, I^2^ = 0%).

### Publication bias

No significant publication bias was observed for the primary outcome of viral suppression efficacy, as assessed in a series of funnel plots (Figure S1a–e). Furthermore, there were no obvious publication bias for secondary outcomes of serological responses (Figure S2a&b) and safety assessment (Figure S3b–d), with the exception of an apparently significant publication bias in overall AE (Figure S3a).

**Figure 5:**
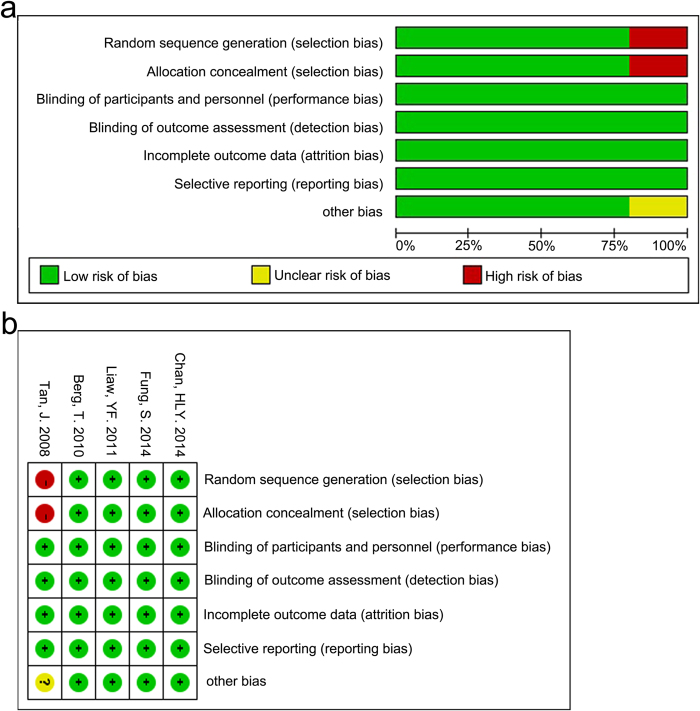
Risk of bias in all included studies was assessed using the Cochrane Collaboration’s tool. (**a**) Risk of bias graph: each risk of bias item was presented as percentages in all included studies; (**b**) Risk of bias summary: each risk of bias item was presented in each included study.

The risk of bias across all included studies was assessed using the Cochrane Collaboration’s tool, as shown in Fig. 5a,b.

## Discussion

Long-term monotherapy of nucleotides analogues has been limited due to a high rate of resistance in the treatment of CHB infection, such as LAM resistance^5^,^7^ and ADV-resistance mutations^8^. A recent meta-analysis^24^ indicated that the combined therapy of ADV and LAM did not show obvious therapeutic superiority when administered in a short duration, but had a great advantage over monotherapy in term of both virological and biochemical responses after the long-term administration. Importantly, current treatment guidelines recommend rescue therapy based on switching to a more potent drug or a combination of two drugs with different resistance profiles^5^.

TDF has been shown to provide superior antiviral efficacy in CHB patients^8^,^11^,^15^, and to exert significant viral suppression efficacy with a favorable safety profile in patients with LAM-resistance even after the failure of ADV therapy^12^,^13^. To pursue a higher efficacy of CHB therapy, the combination therapy of TDF and other drugs were performed. Notably, the *in vitro* study demonstrated that the combination of FTC and TDF could induce a synergistic effect on anti-HBV activity^18^. However, an extensive debate on the therapeutic efficacy of TDF alone and FTC/TDF combination in clinical practices still exists. Tan *et al.*^19^ reported that TDF monotherapy was effective for patients with virologic breakthrough or suboptimal response to ADV, while FTC/TDF combination should be considered in patients with ADV-resistance. Chan *et al.*^22^ conducted a double-blind clinical trial, which indicated FTC/TDF combination provided better viral suppression than TDF alone in HBeAg positive CHB patients with high viral loads. In contrast, some pivotal clinical trials showed similar therapeutic efficiency in CHB patients between the two treatment groups^10^,^20^,^21^.

Therefore, we performed a meta-analysis to comprehensively assess the therapeutic effects of TDF alone and FTC/TDF combination in CHB patients. We found that the FTC/TDF combination treatment provided better viral suppression efficacy versus TDF alone treatment in CHB patients with nucleos(t)ide-naïve treatment history, while both treatments presented a similar viral suppression efficacy in CHB patients with nucleos(t)ide treatment history. Moreover, analysis of the secondary outcomes analysis indicated that there were no significant differences in serological responses including HBeAg loss and HBeAg seroconversion between the treatment groups with TDF alone and FTC/TDF combination at different time points after therapy. Furthermore, both treatments groups showed similar safety outcomes with regard to overall AE, drug-related AE, SAE and drug-related SAE.

In the included five studies, four studies^10^,^20^,^21^,^22^ with 601 patients were multicenter, double-blind, RCTs, and the quality assessment of these four studies was high and persuasive. Only one study with 13 patients (10 patients treated with TDF versus 3 treated with FTC/TDF) was an open-labeled observation, which might contribute to heterogeneity. In the initial meta-analysis of the five studies, we found no significant difference in viral suppression efficacy between the treatment groups with TDF alone and FTC/TDF combination after 12 weeks and 24 weeks; however, an obvious difference were observed after 48 weeks. Notably, no obvious difference between the two treatment groups recurred after 96 weeks with statistically significant heterogeneity (I^2^ = 69%). Thus, the total analysis was stratified by the nucleos(t)ide drug treatment history. In both subgroups, a total of 488 patients in four studies^10^,^19^,^20^,^21^ had a treatment history of nucleos(t)ide drugs, while another RCT study^22^ enrolled 126 nucleos(t)ide-naïve patients with high HBV viral load. Consequently, a serial of funnel plots also demonstrated that there was no significant publication bias for the primary outcome of viral suppression efficacy.

Several factors may contribute to heterogeneity among clinical trials. First, the long-term use of different nucleos(t)ide drugs including LAM, ADV and both in combination probably resulted in heterogeneity, which might result in variation in the risks of unfavorable clinical outcomes. Only one study involved nucleos(t)ide-naïve patients with high HBV load^22^. Second, patient variation in the immune response may affect the viral suppression efficacy of the two treatments. At the time of treatment initiation, the patients in one study^22^ were in the immune-tolerant phase, while the patients in the other four studies^10^,^19^,^20^,^21^ were in the immune-active status. Third, the variation in HBV genotypes may affect the viral suppression efficacy of the two treatments. In one study^22^, genotype B patients were predominant (58%), but not in the other four studies (10%, 0%, 13% and 24%,)^10^,^19^,^20^,^21^. Finally, gender may be a crucial factor influencing viral suppression efficacy of the two treatments. A pivotal RCT study^22^ has indicated that female sex is associated with a favorable response to antiviral treatment, independent of the treatment received, although the reasons for this association remain unclear. In one study^22^, females represented 51% of the patients, but in the other four studies females represented 24%, 15%, 25% and 14% of the patients^10^,^19^,^20^,^21^.

This study provided direct evidence that the FTC/TDF combination treatment showed better viral suppression efficacy versus TDF alone treatment in CHB patients with nucleos(t)ide-naïve treatment history, suggesting that the treatment of FTC and TDF combination should be prior consideration in the nucleos(t)ide-naïve treatment CHB patients. However, for CHB patients with nucleos(t)ide treatment history, both treatments provided similar viral suppression efficacy and safety, which indicated that TDF monotherapy should be prior consideration from the aspect of economic cost.

Interestingly, previous researches had also indicated a similar phenomenon in therapeutic efficacy of other nucleotides analogues for CHB patients. A meta-analysis of five studies (328 patients in total) conducted by Liu *et al.*^25^ showed that, in CHB patients with nucleos(t)ide-naïve treatment history, LAM plus ADV combination therapy showed better efficacy than ETV monotherapy in term of viral suppression efficacy, biochemical response and HBeAg seroconversion. However, a meta-analysis of eight studies (696 patients in total)conducted by Huang *et al.*^26^ found that, in CHB patients with lamivudine treatment history (and LAM resistance), there was no significant difference between the LAM plus ADV combination therapy group (355 patients) and the ETV monotherapy group (341 patients) in terms of HBV suppression efficacy, HBeAg loss, HBeAg seroconversion and overall AE. In our meta-analysis, although only one study involving the nucleos(t)ide-naïve treatment CHB patients was enrolled, this study involved 126 patients and was a high-quality RCT. Notably, therapeutic efficacies of FTC/TDF combination treatment versus TDF alone treatment in our study were consistent with those of LAM plus ADV combination therapy versus ETV monotherapy in CHB patients with nucleos(t)ide-naïve treatment history^25^ or CHB patients with nucleos(t)ide treatment history^26^. Thus, we speculated that this phenomenon was not an anomaly and our results were credible and meaningful.

In conclusion, this study demonstrated that both TDF monotherapy and FTC/TDF combination were efficacious and safe in CHB patients. Importantly, the FTC/TDF combination treatment showed better viral suppression efficacy versus TDF alone in CHB patients with nucleos(t)ide-naïve treatment, while both treatments provided similar viral suppression efficacy in CHB patients with nucleos(t)ide treatment history. This comprehensive analysis indicates a relatively clear approach to the achievement of viral suppression in CHB patients, which may provide a better choice for viral suppression and long-term survival of the CHB patients.

## Methods

### Literature search

A computerized search was performed by two independent investigators (G-Y.C. and H-Y.D.) in PubMed/Medline, EMBASE, the Cochrane Library, and Web of Science databases to identify relevant articles published between 1995 and October 20, 2014. The following terms was used for literature search: “chronic hepatitis B”, “hepatitis B virus”, “HBV”, “Tenofovir disoproxil fumarate”, “TDF”, “Emtricitabine” and “FTC” with all possible combinations. Based on these parameters, we filtered out all the eligible articles and scanned their reference lists for additional available studies.

### Inclusion and exclusion criteria

To adhere to the high standards required of meta-analyses, all of the selected articles were collected and reviewed independently by two reviewers (G-Y.C. and H-Y.D.) to determine their eligibility for detailed analysis. The included studies were required fulfill the following criteria: (1) RCTs with publication in English; (2) adult patients with chronic hepatitis B, (3) using TDF plus FTC and TDF alone as treatments; (4) providing valid data directly or data that could be calculated indirectly; and (5) the study with the highest quality assessment was enrolled when same trials were reported more than once.

Review articles without original data, abstracts, editorials and letters to the editor, expert opinions, case reports, and studies lacking control groups were excluded. Studies and data were also excluded if: (1) conducted in animals or cell lines; (2) the outcomes or parameters of patients were not clearly reported (e.g. omitting standard deviations (SDs) (3) conference records; (4) absence of related data required for necessary analysis; (5) overlapping articles. If data were missing from a study, the investigator was contacted to provide the missing data if possible. The quality of each study was assessed by a Jadad score^23^ and criteria based on those reported by Juni *et al.*^27^.

### Outcomes

The primary outcome was the efficacy of viral suppression. The primary efficacy end-point was the proportion of CHB patients achieving HBV DNA <69 IU/mL after treatment with TDF alone or FTC/TDF combination. This level was chosen because it concurred with the primary end-point of the pivotal studies of TDF^15^. The secondary outcomes were mainly serological responses (HBeAg loss and HBeAg seroconversion) and safety assessment including all adverse events (AE), drug-related AE, serious adverse events (SAE) and drug-related SAE.

### Statistical analysis

This meta-analysis was performed using the Review Manager (RevMan) software (version 5.3.4; Cochrane collaboration, http://tech.cochrane.org/revman/download). The meta-analysis compared the efficacy of viral suppression, serological responses and safety assessment at 12, 24, 48, 96 and 192 weeks after treatments with TDF alone and FTC/TDF combination in CHB patients using odds ratios (OR) and 95% confidence intervals (95% CI) that were calculated using either a fixed-effects or a random-effects model.

Heterogeneity among the outcomes of enrolled studies was assessed with Chi-square based Q statistical tests. The I^2^ statistic was calculated to quantify the total variation consistent with inter-study heterogeneity, ranging from 0% to 100%. Heterogeneity was significant and unacceptable when I^2^ was greater than 50%. At P ≥ 0.05, heterogeneity was considered no statistically significance, and the fixed effect model was used in the analysis; at P < 0.05, heterogeneity was considered statistically significant, and the random effect model was used in the analysis.

The funnel plots were generated utilizing Egger’s test and Begg’s test to examine the risk of potential publication bias. Trim and fill analyses were then used to evaluate the stability of the meta-analysis results if the plots were asymmetrical.

## Additional Information

**How to cite this article**: Cui, G. *et al.* Comparative Meta-Analysis of Tenofovir Disoproxil Fumarate versus Emtricitabine and Tenofovir Disoproxil Fumarate as Treatments for Patients with Chronic Hepatitis B. *Sci. Rep.* **5**, 11854; doi: 10.1038/srep11854 (2015).

## Supplementary Material

Supplementary Information

## Figures and Tables

**Table 1:** Characteristics of studies included in the meta-analysis.

**Study (year)**	**Study type**	**Location**	**Treatment**	**No. of patients**	**Mean age(range)**	**Gender (M/F)**	**HBV DNA (log10 copies/mL)**	**HBeAg positive**	**Normal ALT (No.)**	**Nucleos(t)ide Drug history**	**Study duration**	**Jadad score**
Tan, J(2008)	NonRCT	United States	TDFFTC/TDF	103	47.7 (35-72)61.6 (57–66)	11/2^b^	4.4-105.0–6.3	11/13^d^	1/100/3	ADV	27months	1
Liaw, YF(2011)	RCT	39 study centersin 5 region	TDFFTC/TDF	4545	52 (48–57)^a^50 (42–58)^1^	37/840/5	4.7–6.6^a^4.5–7.3^a^	14/4518/45	18/4518/45	19/45^e^ 9/45^f^17/45^e^ 10/45^f^	48weeks	4
Berg, T(2010)	RCT	28 study centersin 4 regions	TDFFTC/TDF	5352	40 (18–59)39 (19–59)	38/1542/10	3.41–9.572.23–9.47	38/5339/52	26/5326/52	ADV	48weeks	4
Chan, HLY(2014)	RCT	34 study centersin 11 countries	TDFFTC/TDF	6462	33 (18–62)33 (18–58)	31/3331/31	8.02–10.227.79–9.87	63/6462/62	60/6456/62	Naïve	192weeks	4
Fung, S(2014)	RCT	62 study centersin 3 regions	TDFFTC/TDF	141139	47.1 (unclear)46.3 (unclear)	104/37107/32	5.64 ± 1.83^c^5.77± 1.97^c^	65/14168/139	62/14156/139	LAM	96weeks	4

^a^median interquartile range;

^b^the male and female in total patients;

^c^mean ± SD;

^d^HBeAg positive patients in all patients;

^e^Drug history of LAM, Previous CHB treatment experience, Lamivudine >6 months;

^f^Drug history of ADV.

## References

[b1] Fattovich G. Natural history and prognosis of hepatitis B. Semin Liver Dis 23, 47–58 (2003). 12616450 10.1055/s-2003-37590

[b2] Ganem D. & Prince A. M. Hepatitis B virus infection–natural history and clinical consequences. N Engl J Med 350, 1118–1129 (2004). 15014185 10.1056/NEJMra031087

[b3] World Health Organization. Hepatitis B (Updated March 2015). Available from: http://www.who.int/mediacentre/factsheets/fs204/en/. Date of access: 26/03/2015.

[b4] Si-Ahmed S. N. *et al.* Efficacy and tolerance of a combination of tenofovir disoproxil fumarate plus emtricitabine in patients with chronic hepatitis B: a European multicenter study. Antiviral research 92, 90–95 (2011). 21767570 10.1016/j.antiviral.2011.07.003

[b5] EASL clinical practice guidelines: Management of chronic hepatitis B virus infection. J Hepatol 57, 167–185 (2012). 22436845 10.1016/j.jhep.2012.02.010

[b6] Liaw Y. F. *et al.* Asian-Pacific consensus statement on the management of chronic hepatitis B: a 2008 update. Hepatol Int 2, 263–283 (2008). 19669255 10.1007/s12072-008-9080-3 PMC2716890

[b7] Leung N. W. *et al.* Extended lamivudine treatment in patients with chronic hepatitis B enhances hepatitis B e antigen seroconversion rates: results after 3 years of therapy. Hepatology 33, 1527–1532 (2001). 11391543 10.1053/jhep.2001.25084

[b8] Hadziyannis S. J. *et al.* Long-term therapy with adefovir dipivoxil for HBeAg-negative chronic hepatitis B for up to 5 years. Gastroenterology 131, 1743–1751 (2006). 17087951 10.1053/j.gastro.2006.09.020

[b9] Peters M. G. *et al.* Adefovir dipivoxil alone or in combination with lamivudine in patients with lamivudine-resistant chronic hepatitis B. Gastroenterology 126, 91–101 (2004). 14699491 10.1053/j.gastro.2003.10.051

[b10] Berg T. *et al.* Tenofovir is effective alone or with emtricitabine in adefovir-treated patients with chronic-hepatitis B virus infection. Gastroenterology 139, 1207–1217 (2010). 20600025 10.1053/j.gastro.2010.06.053

[b11] Marcellin P. *et al.* Regression of cirrhosis during treatment with tenofovir disoproxil fumarate for chronic hepatitis B: a 5-year open-label follow-up study. Lancet 381, 468–475 (2013). 23234725 10.1016/S0140-6736(12)61425-1

[b12] van Bommel F. *et al.* Tenofovir for patients with lamivudine-resistant hepatitis B virus (HBV) infection and high HBV DNA level during adefovir therapy. Hepatology 44, 318–325 (2006). 16871563 10.1002/hep.21253

[b13] van Bommel F. *et al.* Long-term efficacy of tenofovir monotherapy for hepatitis B virus-monoinfected patients after failure of nucleoside/nucleotide analogues. Hepatology 51, 73–80 (2010). 19998272 10.1002/hep.23246

[b14] Patterson S. J. *et al.* Tenofovir disoproxil fumarate rescue therapy following failure of both lamivudine and adefovir dipivoxil in chronic hepatitis B. Gut 60, 247–254 (2011). 21036792 10.1136/gut.2010.223206

[b15] Marcellin P. *et al.* Tenofovir disoproxil fumarate versus adefovir dipivoxil for chronic hepatitis B. N Engl J Med 359, 2442–2455 (2008). 19052126 10.1056/NEJMoa0802878

[b16] Borroto-Esoda K., Parkin N. & Miller M. D. A comparison of the phenotypic susceptibility profiles of emtricitabine and lamivudine. Antivir Chem Chemother 18, 297–300 (2007). 18046962 10.1177/095632020701800505

[b17] Lim S. G. *et al.* A double-blind placebo-controlled study of emtricitabine in chronic hepatitis B. Arch Intern Med 166, 49–56 (2006). 16401810 10.1001/archinte.166.1.49

[b18] Zhu Y., Curtis M., Qi X., Miller M. D. & Borroto-Esoda K. Anti-hepatitis B virus activity *in vitro* of combinations of tenofovir with nucleoside/nucleotide analogues. Antivir Chem Chemother 19, 165–176 (2009). 19374144 10.1177/095632020901900404

[b19] Tan J. *et al.* Tenofovir monotherapy is effective in hepatitis B patients with antiviral treatment failure to adefovir in the absence of adefovir-resistant mutations. Journal of hepatology 48, 391–398 (2008). 18199519 10.1016/j.jhep.2007.09.020

[b20] Fung S. *et al.* Randomized comparison of tenofovir disoproxil fumarate vs emtricitabine and tenofovir disoproxil fumarate in patients with lamivudine-resistant chronic hepatitis B. Gastroenterology 146, 980–988 (2014). 24368224 10.1053/j.gastro.2013.12.028

[b21] Liaw Y. F. *et al.* Tenofovir disoproxil fumarate (TDF), emtricitabine/TDF, and entecavir in patients with decompensated chronic hepatitis B liver disease. Hepatology 53, 62–72 (2011). 21254162 10.1002/hep.23952

[b22] Chan H. L. *et al.* Effects of tenofovir disoproxil fumarate in hepatitis B e antigen-positive patients with normal levels of alanine aminotransferase and high levels of hepatitis B virus DNA. Gastroenterology 146, 1240–1248 (2014). 24462735 10.1053/j.gastro.2014.01.044

[b23] Jadad A. R. *et al.* Assessing the quality of reports of randomized clinical trials: is blinding necessary? Control Clin Trials 17, 1–12 (1996). 8721797 10.1016/0197-2456(95)00134-4

[b24] Chen Y. & Ju T. Comparative meta-analysis of adefovir dipivoxil monotherapy and combination therapy of adefovir dipivoxil and lamivudine for lamivudine-resistant chronic hepatitis B. Int J Infect Dis 16, e152–158 (2012). 22226087 10.1016/j.ijid.2011.11.006

[b25] Liu F. *et al.* Efficacy and resistance in de novo combination lamivudine and adefovir dipivoxil therapy versus entecavir monotherapy for the treatment-naive patients with chronic hepatitis B: a meta-analysis. Virology journal 11, 59 (2014). 24673792 10.1186/1743-422X-11-59 PMC3986697

[b26] Huang Z. B. *et al.* Comparison of the efficacy of Lamivudine plus adefovir versus entecavir in the treatment of Lamivudine-resistant chronic hepatitis B: a systematic review and meta-analysis. Clinical therapeutics 35, 1997–2006 (2013). 24238791 10.1016/j.clinthera.2013.10.002

[b27] Juni P., Altman D. G. & Egger M. Systematic reviews in health care: Assessing the quality of controlled clinical trials. BMJ 323, 42–46 (2001). 11440947 10.1136/bmj.323.7303.42 PMC1120670

