# Supplementary material for: Comparative Meta-Analysis of Tenofovir Disoproxil Fumarate versus Emtricitabine and Tenofovir Disoproxil Fumarate as Treatments for Patients with Chronic Hepatitis B
Source: Sci Rep. 2015 Jul 13;5:11854. doi: 10.1038/srep11854 (PMC4499796; doi:10.1038/srep11854)
Supplement: Supplementary Information [file srep11854-s1.doc]

**Comparative Meta-Analysis of Tenofovir Disoproxil Fumarate versus Emtricitabine and Tenofovir Disoproxil Fumarate as Treatments for Patients with Chronic Hepatitis B**

Guangying Cui^1,2^ , Xuejun Xu^3^, Hongyan Diao^1,2^

^1^ State Key Laboratory for Diagnosis and Treatment of Infectious Diseases, The First Affiliated Hospital, School of Medicine, Zhejiang University, Hangzhou, Zhejiang, 310003, China

^2^ Collaborative Innovation Center for Diagnosis and Treatment of Infectious Diseases, Hangzhou, China

^3^ Department of Orthodontics, Affiliated Stomatology Hospital, School of Medicine, Zhejiang University, Hangzhou, Zhejiang, 310006, China

**Corresponding author:** Hongyan Diao or Xuejun Xu

State Key Laboratory for Diagnosis and Treatment of Infectious Diseases, The First Affiliated Hospital, School of Medicine, Zhejiang University, Hangzhou, Zhejiang, 310003, China. Tel&Fax: 86-571-87236446. E-mail: diaohy@zju.edu.cn

or Department of Orthodontics, Affiliated Stomatology Hospital, School of Medicine, Zhejiang University, Hangzhou, Zhejiang, 310006, China. E-mail: xxj4007@163.com

**Supporting Information**

**
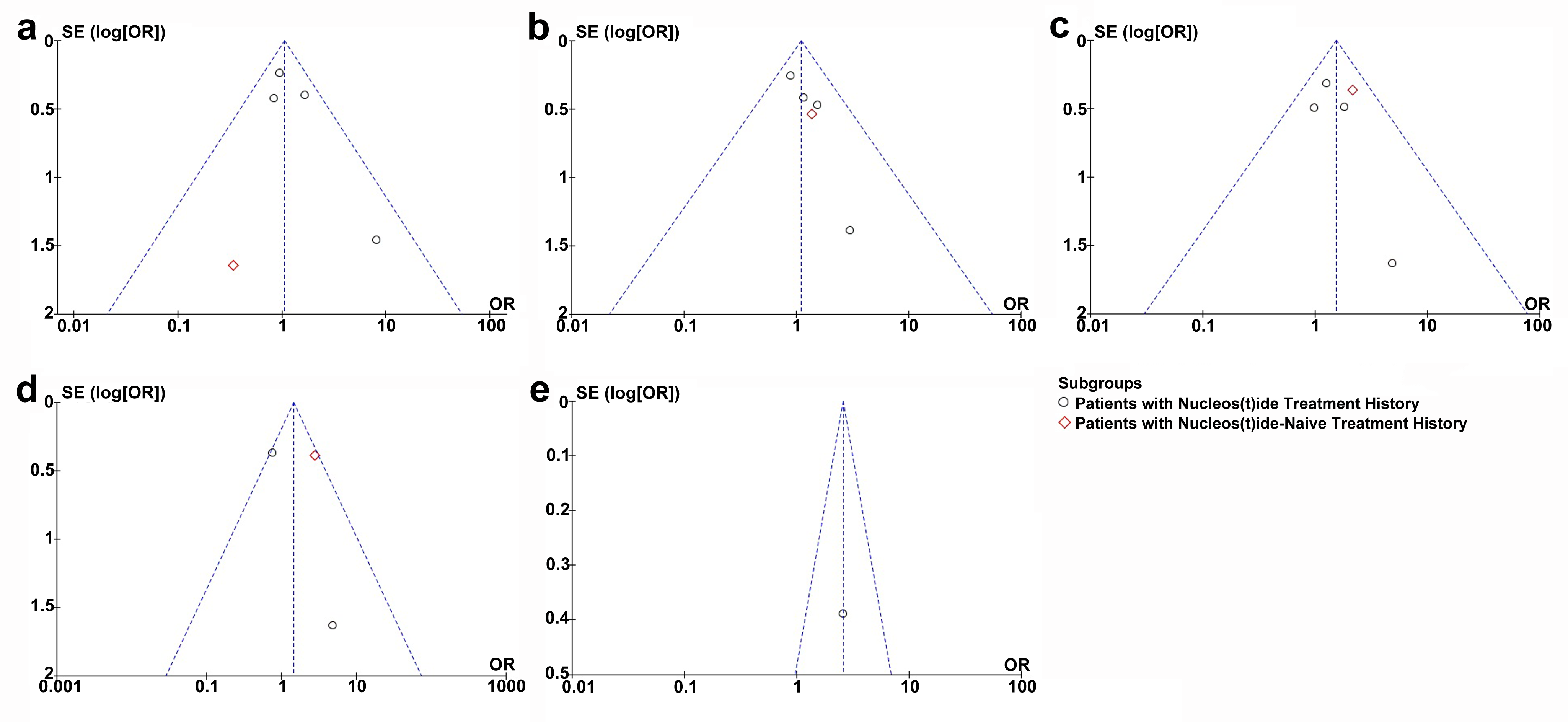
**

**Figure S1** **Funnel plots were made by Egger’s test and Begg’s test to assess publication bias for the primary outcome.** A serial of funnel plots showed no significant publication bias for the primary outcome of viral suppression efficacy in CHB patients with nucleos(t)ide-(or naïve) treatment history after (a) 12 weeks, (b) 24 weeks, (c) 48 weeks, (d) 96 weeks and (e) 192 weeks of treatments with TDF alone and FTC/TDF combination, respectively.


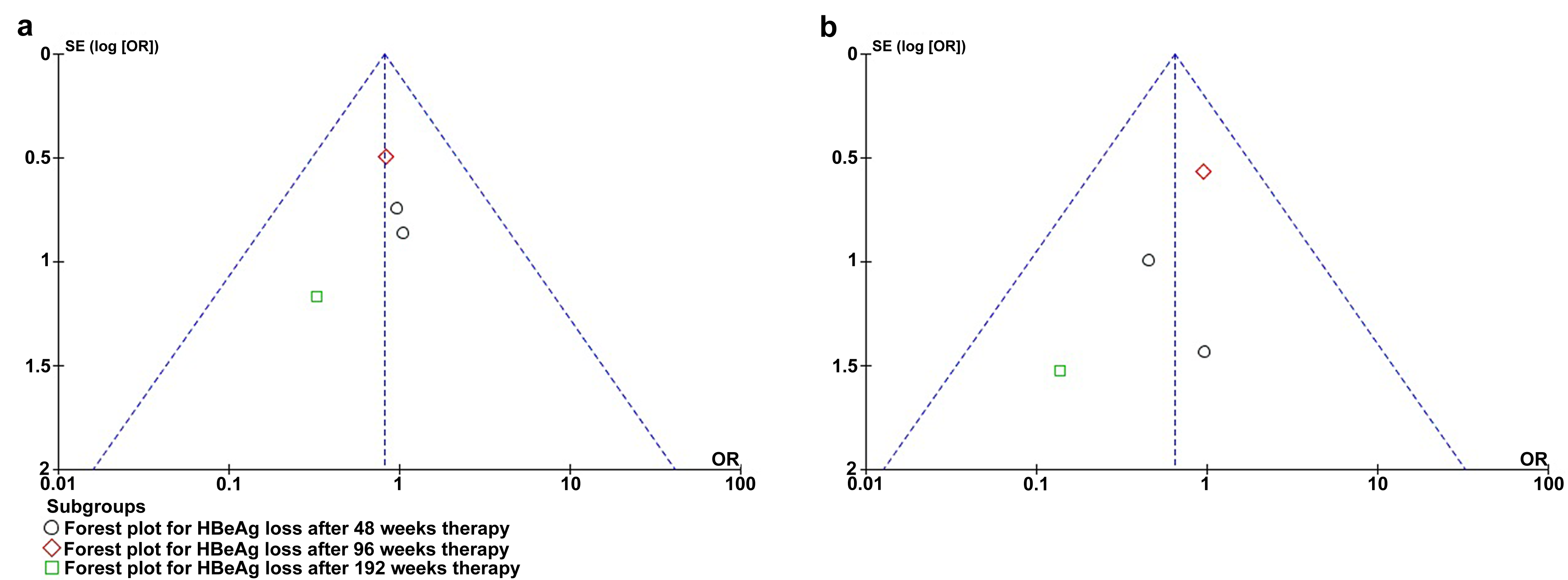


**Figure S2** **Funnel plots were made by Egger’s test and Begg’s test to assess publication bias for the secondary outcomes: serological responses.** Funnel plots showed no obvious publication bias for (a) HBeAg loss and (b) HBeAg seroconversion in CHB patients after 48, 96 and 192 weeks of treatments with TDF alone and FTC/TDF combination, respectively.


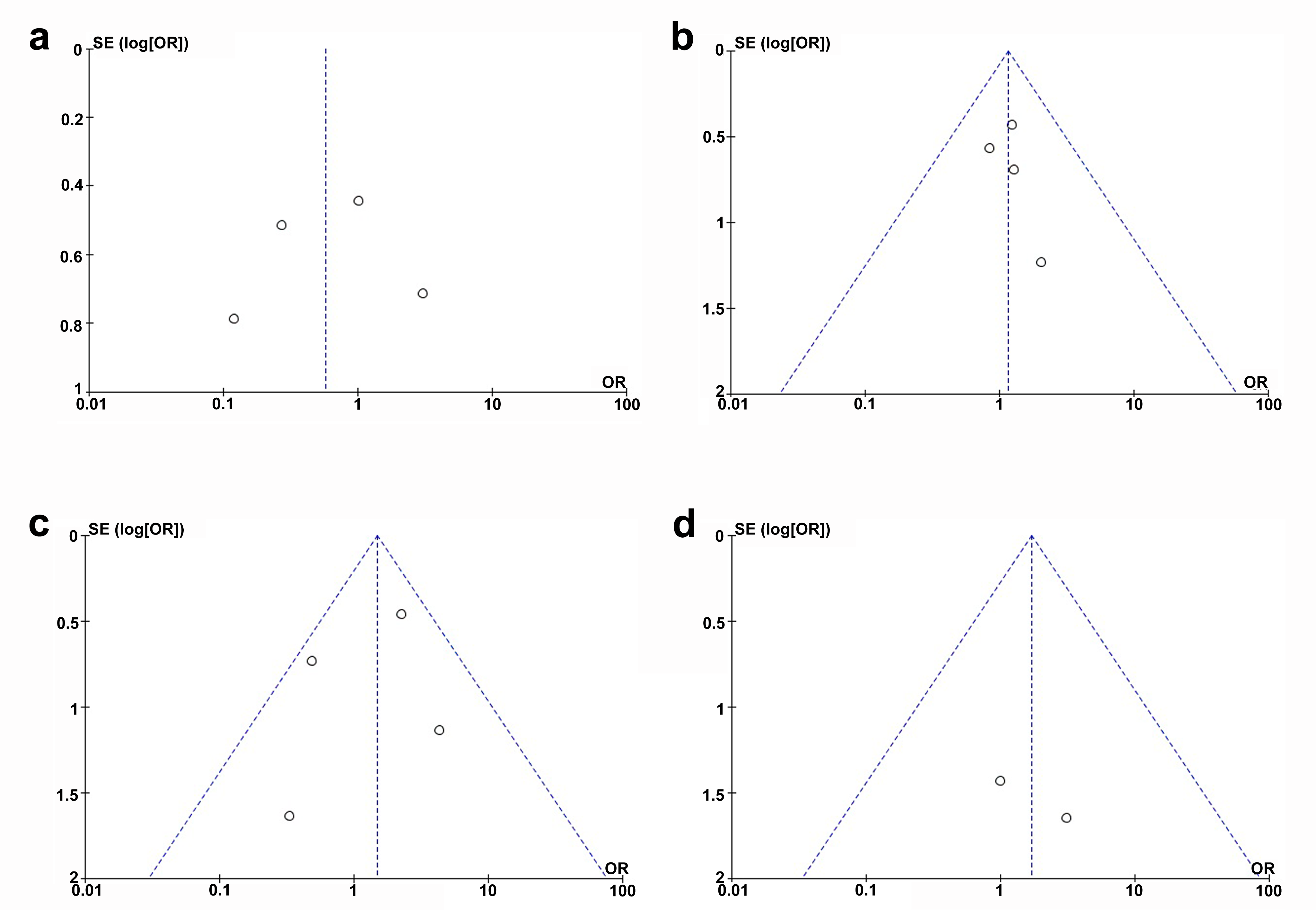


**Figure S3** **Funnel plots were made by Egger’s test and Begg’s test to assess publication bias for the secondary outcomes: safety assessment.** Funnel plots showed no significant publication bias for comparison of (b) drug-related AE, (c) SAE and (d) drug-related SAE in CHB patients after treatments with TDF alone and FTC/TDF combination, respectively, except for a seemingly significant publication bias in (a) overall AE.

(TIF)
